# Supplementary material for: Saccharomyces cerevisiae TAD1 Mutant Strain As Potential New Antimicrobial Agent: Studies on Its Antibacterial Activity and Mechanism of Action
Source: Microorganisms. 2025 Dec 15;13(12):2848. doi: 10.3390/microorganisms13122848 (PMC12735431; doi:10.3390/microorganisms13122848)
Supplement: Supplementary file 1 [file microorganisms-13-02848-s001.zip › microorganisms-4015949-supplementary.pdf]

1.Table S1. Primer sequences used for quantitative real-time PCR.

Gene	Primers sequence (5' to 3')
Occludin	F:TTGAAAGTCCACCTCCTTACAGA
	R:CCGGATAAAAAGAGTACGCTGG
Claudin-1	F:GGGGACAACATCGTGACCG
	R:AGGAGTCGAAGACTTTGCACT
ZO-1	F:GCCGCTAAGAGCACAGCAA
	R:TCCCCACTCTGAAAATGAGGA
TNF-α	F:CCGAGAGCGGAAGTGTGTG
	R:TGTAACTGTGGTTTTGGTCTTCA
IL-10	F:GCTCTTACTGACTGGCATGAG
	R:CGCAGCTCTAGGAGCATGTG
IL-6	F:CTGCAAGAGACTTCCATCCAG
	R:AGTGGTAGACAGGTCTGTTGG
NF-κB	F:ATGGCAGACGATGATCCCTAC
	R:TGTTGACAGTGGTATTTCTGGTG
β-actin	F:GGCTGTATTCCCCTCCATCG
	R:CCAGTTGGTAACAATGCCATGT

F: Forward primer; R: Reverse primer.

2. Table S2. Primer sequences used for quantitative real-time PCR.

Gene	Primers sequence (5' to 3')
fliC	F:CCATCATCTCCGCCCAGTTT
	R:TACGCTGCGGATGTGAATGA
fimH	F:CTGATGGGCTGGTCGGTAAA
	R:CGCTGGTGGTAGGAAATGGA
csgA	F:GGCAGGTGTTGTTCCTCAGT
	R:CTGTCATCTGAGCCCTGACC
csgD	F:CGTAAAGTAGCATTCGCCGC
	R:GATTACCCGTACCGCGACAT
flhC	F:GGCTGGTGAGCGTGGGTAATA
	R:AGTGCCCGCAAGCAGAAGAAG
flhD	F:CGTCCGCTATGTTTCGTCTC
	R:CTGAGTAATCGTCTGGTGGC
16sRNA	F:ACTCCTACGGGAGGCAGCAG
	R:ATTACCGCGGCTGCTGG

F: Forward primer; R: Reverse primer.

3. LC-MS non-targeted metabolomics analysis method.

I. Project Information

1.1 Introduction to the Experiment

This experiment commenced with the precise weighing of a defined sample mass. Following grinding (where required for tissue or cellular specimens), extraction of metabolites was conducted in a low-temperature environment using extraction solvent. Subsequently, centrifugation yielded the supernatant metabolic product solvent for liquid chromatography-mass spectrometry analysis. Raw data were processed using the metabolomics software Progenesis QI v3.0 (Waters Corporation, Milford, USA) for peak extraction, alignment, and identification. This yielded a data matrix containing retention times, peak areas, mass-to-charge ratios, and identification information, which was utilised for subsequent processing and bioinformatics analysis.

1.2 Sample Information

This experiment received a total of 24 samples; detailed information is provided in the sample information sheet.

II. Experimental Methods

2.1 Sample Preparation

1. Pipette precisely 200 μL of sample into a 1.5 mL centrifuge tube;
2. Add 800 μL extraction solvent (methanol:acetonitrile = 1:1 (v:v)), containing four internal standards (L-2-chlorophenylalanine (0.02 mg/mL) etc.);
3. Vortex for 30 seconds, then perform low-temperature ultrasonic extraction for 30 minutes (5°C, 40 kHz);
4. Allow the sample to stand at -20°C for 30 minutes;
5. Centrifuge for 15 minutes (13,000g, 4°C), transfer the supernatant, and evaporate to dryness under nitrogen;
6. Add 120 μL of resuspension buffer (acetonitrile:water = 1:1) to resuspend;
7. Vortex for 30 seconds, followed by low-temperature ultrasonic extraction for 5 minutes (5°C, 40 kHz);
8. Centrifuge for 10 min (13,000 g, 4°C), transfer supernatant to an injection vial with an insert tube for instrument analysis;
9. Additionally, transfer 20 μL supernatant from each sample, pool, and use as quality control samples.

2.2 LC-MS Detection

The instrument platform employed for this LC-MS analysis was Thermo Fisher Scientific's ultra-high-performance liquid chromatography tandem Fourier transform ion-focusing mass spectrometry system, the UHPLC-Q Exactive HF-X.

Chromatographic conditions: The column was an ACQUITY UPLC HSS T3 (100 mm × 2.1 mm i.d., 1.8 μm; Waters, Milford, USA); mobile phase A was 95% water + 5% acetonitrile (containing 0.1% formic acid), mobile phase B was 47.5% acetonitrile + 47.5% isopropanol + 5% water (containing 0.1% formic acid), injection volume was 3 μL, and column temperature was 40° C.

Mass spectrometry conditions: Samples were analysed via electrospray ionisation, with mass signals acquired in both positive and negative ion scanning modes. Specific parameters are detailed in the table below:

Table 1 Mass Spectrometry Parameters

Description	Parameters
Scan type（m/z）	70-1050
Sheath gas flow rate（arb）	50
Aux gas flow rate（arb）	13
Heater temp（℃）	425
Capillary temp（℃）	325
Spray voltage（+）（V）	3500
Spray voltage（-）（V）	-3500
S-Lens RF Level	50
Normalized collision energy（%）	20,40,60
Resolution（Full MS）	60000
Resolution（MS 2）	7500

2.3 Quality Control

Quality control (QC) samples are prepared by mixing equal volumes of the extraction solutions from all samples. Each QC sample has the same volume as the analytical samples and is processed and analysed using identical methods. During instrumental analysis, one QC sample is inserted for every 5 to 15 analytical samples to assess the overall stability of the testing process.

III. Summary

This study incorporated 27 samples for LC-MS/MS analysis. Following instrumentation, raw data underwent peak detection, extraction, alignment, and integration using Progenesis QI (Waters Corporation, Milford, USA). Substance annotation was performed using the HMDB database (http://www.hmdb.ca/), Metlin (https://metlin.scripps.edu/), and Meiji's proprietary database. To eliminate or minimise errors arising from experimental and analytical processes, pre-processing of the qualified data was required. This involved removing features with missing values exceeding 20% within each group in the raw data, followed by imputation of missing values using the minimum value across all samples. The response intensities of the sample mass spectrometry peaks were normalised using a sum-data normalisation method, yielding a normalised data matrix. Simultaneously, variables with a relative standard deviation (RSD) exceeding 30% in QC samples were removed, and log10 transformation was applied to yield the final data matrix for subsequent analysis. Statistical analyses including PCA and OPLS-DA were performed using the ropls package (Version 1.6.2) in R. Metabolite annotation was conducted via the HMDB and KEGG databases (https://www.kegg.jp/kegg/pathway.html), whilst pathway enrichment analysis was executed using the scipy.stats Python package.
